# The Gut Microbiota-Immunity Axis in ALS: A Role in Deciphering Disease Heterogeneity?

**DOI:** 10.3390/biomedicines9070753

**Published:** 2021-06-29

**Authors:** Elena Niccolai, Vincenzo Di Pilato, Giulia Nannini, Simone Baldi, Edda Russo, Elisabetta Zucchi, Ilaria Martinelli, Marta Menicatti, Gianluca Bartolucci, Jessica Mandrioli, Amedeo Amedei

**Affiliations:** 1 Department of Clinical and Experimental Medicine, University of Florence, 50134 Florence, Italy; elena.niccolai@unifi.it (E.N.); giulia.nannini@unifi.it (G.N.); simone.baldi@unifi.it (S.B.); edda.russo@unifi.it (E.R.); 2 Department of Surgical Sciences and Integrated Diagnostics (DISC), University of Genoa, 16132 Genoa, Italy; vincenzo.dipilato@unige.it; 3 Department of Biomedical, Metabolic and Neural Sciences, University of Modena and Reggio Emilia, 41125 Modena, Italy; elibettizucchi@gmail.com; 4 Neurology Unit, Department of Neuroscience, Azienda Ospedaliero Universitaria di Modena, 41125 Modena, Italy; martinelli.ilaria88@gmail.com; 5 Department of Neurosciences, Psychology, Drug Research and Child Health Section of Pharmaceutical and Nutraceutical Sciences, University of Florence, 50139 Florence, Italy; marta.menicatti@unifi.it (M.M.); gianluca.bartolucci@unifi.it (G.B.)

**Keywords:** Amyotrophic Lateral Sclerosis, microbiota, inflammation, cytokines, short chain fatty acids, heterogeneity, motor neuron disease

## Abstract

Amyotrophic Lateral Sclerosis (ALS) is a neurodegenerative disorder with an unknown etiology and no effective treatment, and is characterized by large phenotypic heterogeneity, including variable sites, ages of symptom onset and rates of disease progression. Increasing data support the role of the microbiota-immunity axis in the pathogenesis of neurodegenerative diseases. In the present study, we compared the inflammatory and microbiota profile of ALS patients with different clinical characteristics, with healthy family caregivers. Measuring a panel of 30 inflammatory cytokines in serum and fecal samples, we observed a distinct cytokine profile both at the systemic and intestinal level in patients compared to controls and even in patients with different clinical phenotypes and progression rates. The 16S targeted metagenome analysis revealed slight differences in patients compared to controls as well as in patients with slow progression, marked by the reduction of butyrate-producing bacteria and a decrease of the Firmicutes/Bacteroidetes ratio in ALS. Finally, the short chain fatty acid analysis did not show a different distribution among the groups. If confirmed in a larger number of patients, the inflammatory cytokine profile and the microbial composition could be appropriate biomarker candidates for deciphering ALS heterogeneity.

## 1. Introduction

Amyotrophic Lateral Sclerosis (ALS) is a neurodegenerative disorder characterized by the premature death of motor neurons and an average survival rate of 3–5 years from diagnosis [1]. A wide variety of gene mutations have been discovered for the familial form of the disease (fALS), leading to an impressive genetic heterogeneity. Beside genetics, ALS is characterized by large phenotypic variability, including unpredictable sites, ages of symptom onset and rates of disease progression [2]. This heterogeneity can be seen even among patients carrying the same mutation both between and within families, as well as for the various mutations [3,4]. 

Together with gene mutations that may modify the ALS phenotype [5,6], environmental factors have been postulated to modulate the disease progression [7]. The pathological process in ALS, still relatively unknown, is now recognized as extending beyond motor neurons, so it can be regarded as a multi-cellular/multisystemic disease. In addition, both preclinical and clinical evidence suggest an impacting role of the immune system in governing the ALS process in ALS (review in [8]). Finally, emerging evidence suggest that dysfunction of the gastrointestinal tract may play a role in ALS pathogenesis through modification of the gut microbiota-brain axis. The gut microbiota (GM) is a source of potential central nervous system (CNS) disease-modifying bioactive metabolites, potentially contributing to the pathogenesis of neurological disorders by influencing (i) neuronal transmission, (ii) synaptic plasticity, (iii) myelination and (iv) complex host behaviors [9,10]. Since the intestinal tract is the main point of contact of the host immune system and microorganisms, the microbiota influence on systemic immune function may have a crucial impact in ALS pathogenesis and in the variability of the disease course. GF (germ free) mice and antibiotic-treated mouse models display a broad range of immunological abnormalities including an alteration of density, morphology and maturity of microglia, suggesting that GM can influence both the development and functions of CNS immune cells [11]. Interestingly, the supplementation with short chain fatty acids (SCFAs), like butyrate, restored the microglia density and morphology in mice. Notably, SCFAs, which are dietary fiber’s end-metabolism products mainly produced by Bacteroidetes and Firmicutes [12], are known to mediate regulatory T cells (Tregs). ALS is characterized by the simultaneous activation of distinct lymphocyte subsets of T helper (Th) cells, in detail Th1 and Th17, and a decrease of Tregs [13] that have a protective role, and as demonstrated in both mice and humans, a greater number of Tregs is associated with slow disease progression [14,15]. Then, it is conceivable that alterations of microbial metabolites may affect ALS patients by acting directly on CNS cells and/or indirectly through immune system modulation. 

To date, the available studies on the GM structure and function in ALS patients have reported contrasting conclusions [16]. Independent research groups documented differences in microbiota composition of ALS patients compared to controls, marked by a lower Firmicutes/Bacteroidetes (F/B) ratio and differential abundances of specific genera (in particular *Ruminococcus*) [17,18,19], while others have claimed not to find substantial taxonomic and metagenomic differences [20,21]. There can be many explanations for these discrepancies, such as limited patient numbers and the presence of confounding factors (diet, secondary disease effects, dysphagia, etc.), or the large heterogeneity (genetic, phenotypic and pathophysiologic) that characterize ALS disease. In this scenario, we have explored for the first time the gut microbiota and its SCFA composition and the intestinal and systemic inflammatory response in ALS patients compared with their healthy family caregivers.

## 2. Materials and Methods

### 2.1. Study Population

ALS patients were recruited by the tertiary ALS Center in Modena, during their regular follow-up at the Center. According to the Revised El Escorial Criteria (EEC-R), patients could be included in the study if they had a diagnosis of definite (EEC-R0), clinically probable (EEC-R1), probable-laboratory-supported (EEC-R2) possible (EEC-R3), sporadic or familial ALS [22]. Furthermore, patients had to be between 18 and 80 years old, have a BMI ≥ 18, have an onset of symptoms more than 24 months before enrollment, and be capable of providing informed consent. Healthy controls (HC) (selected among the patients’ companions, excluding consanguineous) could be enrolled in the study if they were without known chronic diseases, >18 years old, and were able to understand and give a written informed consent. Exclusion criteria (both for ALS and HC) were the following: presence of dementia or any other condition impairing the ability to understand and sign an informed consent; known organic gastrointestinal disease (such as, but not limited to, malignancy, IBD, chronic diarrhea; celiac disease and/or an important food intolerance (e.g., lactose); autoimmune disorders; severe comorbidities (liver, heart, kidney failure, HIV, TBC or any type of hepatitis); previous complicated gastrointestinal surgery; acute infections requiring antibiotics; travel within the past six months to world areas where the risk of traveler diarrhea is high; use of immunosuppressive or chemotherapy agents; antimicrobial treatment four weeks prior to sample collection; and regular consumption of probiotic products four weeks prior to sample collection. In addition, patients already on percutaneous endoscopic gastrostomy (PEG), noninvasive ventilation (NIV) or intravenous therapy (IV) could not be enrolled in the study. Local Ethics Committee approval (Comitato Etico Provinciale di Modena, study n. 15/17) and an informed written consent were obtained from each participant. Each subject underwent a blood and fecal sample collection. Patients were followed up for at least 30 months; the last observation date was set as 31 December 2020. For each patient the following demographic and clinical data were collected through the Emilia Romagna Register of ALS [23]: sex, age and site of onset (bulbar, upper limb or lower limb, respiratory onset), phenotype (Bulbar ALS (Bp), Classic ALS (Cp), Flail arm/leg ALS (Fp), Upper Motor Neuron predominant ALS (UMNp)) [24], ALSFRS-R score and respiratory function (FVC) [25], genetics, family history, riluzole intake and discontinuation [26], gastrostomy, noninvasive (NIV) or invasive (IV) ventilation support [27], and the cause, place and time of death. Disease progression was considered “slow” if the progression rate, as defined by Kimura and colleagues [28], was ≤ 0.4 points/months; “fast” if progression rate was ≥1 point/month; otherwise disease progression was considered “medium”. 

### 2.2. Sample Collection

Fecal samples were collected in 15 mL falcon tubes and stored at −80 °C. Just before the analysis, each sample was thawed, weight adjusted (to between 0.5–1.0 g) and diluted with a sodium bicarbonate 10 mM solution or PBS (1:1 w/v) respectively, for SCFA analysis or cytokines evaluation. The obtained suspension was briefly stirred in a vortex apparatus, extracted in an ultrasonic bath (for 5 min) and then centrifuged at 5000 rpm (for 10 min). The supernatant (“fecal extract”) was collected and transferred in a 1.5 mL centrifuge tube (sample solution) for the following analysis. Venous blood samples were collected in vacutainers for serum separation and centrifuged at 2000 rpm for 10 min at room temperature. Serum was immediately collected and stored at −20 °C until the analysis, without being thawed and refrozen.

### 2.3. Molecular Inflammatory Response Evaluation

The intestinal and systemic inflammatory response was evaluated in serum and fecal extracts through the testing of 30 cytokines. We used specifically assembled MixMatch Human kits with a Luminex MAGPIX detection system (Affymetrix, Thermo Fisher, Vienna, Austria) and followed the manufacturer’s instructions. More specifically, we analyzed: granulocyte colony-stimulating factor (G-CSF), interferon (IFN)-γ, IFN-α, interleukin (IL)-1 beta, IL-2, IL-4, IL-6, IL-8, IL-9, IL-10, IL-15, IL-17A, IL-27, IL-5, IL-12p70, IL-13, IL-1a, IL-23, IL-18, IL-21, IL-22, monocyte chemotactic protein-1 (MCP-1), macrophage inflammatory protein-1α (MIP-1α), tumor necrosis factor-α (TNFα), vascular endothelial growth factor (VEGF)-A, interferon gamma-induced protein 10 (IP-10), granulocyte-macrophage colony stimulating factor (GM-CSF), soluble intercellular adhesion molecule-1 (sICAM1), P-selectin, and E-selectin. The levels of cytokines were estimated using a 5-parameter polynomial curve (XPonent Software, Luminex Corporation, Austin, TX, USA). The Lower and Upper Limits of Quantification (LLOQ and ULOQ) for the cytokines and chemokines are reported in Supplementary Table S1. Out of range concentrations on the lower or upper end of detection were input with values representing 0.5 times the lowest value or 1.5 times the highest value, respectively.

### 2.4. SCFA Evaluation 

The qualitative and quantitative evaluation of fecal SCFAs and the preparation of standard curves was performed by an Agilent GC-MS system composed of a 5971 single quadrupole mass spectrometer, 5890 gas chromatograph and 7673 autosampler, through our previously described GC-MS method [29]. Briefly, the SCFAs were extracted as follows: an aliquot of 100 µL of fecal extract solution (corresponding to 0.1 mg of stool sample) was added to 50 μL of an ISTDs mixture, 1 mL of tert-butyl methyl ether and 50 µL of 1.0 M HCl solution in a 1.5 mL centrifuge tube. Subsequently, each tube was shaken in a vortex apparatus for 2 min, centrifuged at 10,000 rpm for 5 min, and finally the solvent layer was transferred to an autosampler vial and processed three times.

### 2.5. Microbiota Characterization

Genomic DNA was extracted using the DNeasy PowerLyzer PowerSoil Kit (Qiagen, Hilden, Germany) from frozen (−80 °C) samples, as previously described [30]. Briefly, tissues were added to a bead beating tube and thoroughly homogenized with TissueLyser II (Qiagen) for 5 min at 30 Hz. Total genomic DNA was captured on a silica membrane in a spin column format and subsequently washed and eluted. The quality and quantity of extracted DNA was assessed using the NanoDrop ND-1000 (Thermo Fisher Scientific, Waltham, MA, USA) and the Qubit Fluorometer (Thermo Fisher Scientific, Waltham, MA, USA), respectively. Then, genomic DNA was frozen at −20 °C. Extracted DNA samples were sent to IGA Technology Services (Udine, Italy) where amplicons of the variable V3–V4 region of the bacterial 16 s rRNA gene were sequenced using a paired-end approach (2 × 300 cycles) on the Illumina MiSeq platform, according to the Illumina 16S Metagenomic Sequencing Library Preparation protocol [31]. Sequencing results were analyzed using the QIIME 2 suite (Quantitative Insights Into Microbial Ecology) [32]. Briefly, following raw reads denoising (i.e., error correction, removal of chimeric and singleton sequences, joining of denoised paired-end reads) using DADA2 [33], sequence variants (ASVs) were inferred for each sample amplicon. Taxonomic classification of dereplicated ASVs was performed using a Naive Bayes classifier trained on the SILVA 16S reference database (release 132) (https://www.arb-silva.de/documentation/release-132/; last access: 16 April 2021). Microbial diversity was estimated by evaluating alpha-diversity indices (i.e., Shannon, Simpson, Chao1, evenness indices) and beta-diversity (Bray–Curtis, UniFrac) metrics using specific tools implemented in the QIIME 2 pipeline. 

### 2.6. Statistical Analysis

Statistical analysis was performed with SPSS statistical software (version 27). A non-parametric Mann–Whitney test was used to compare fecal and serum factors levels among patients and controls, while a Kruskal–Wallis test was used to compare their distribution among patients with different clinical characteristics. Cytokine and SCFA levels were expressed as mean ± standard deviation (SD). Regarding the microbiota analysis, differences in the relative abundance of bacterial taxa were evaluated using the Kruskal–Wallis test on pairwise or multiple comparisons. The permutational ANOVA (PERMANOVA) test was applied to beta-diversity distance matrices generated by QIIME2 to test the significance between sample clusters observed following Principal Coordinate Analysis (PCoA); significance was determined through 999 permutations. Spearman correlation coefficients to test between relative abundances of microbial taxa and levels of SCFAs and cytokines, were computed using GraphPad Prism 6 (GraphPad Software, La Jolla, CA, USA), with the Kolmogorov–Smirnov test used to assess the normal distribution of variables. *p* values of less than 0.05 were considered statistically significant.

## 3. Results

### 3.1. Patients

Nineteen consecutive ALS patients and nine healthy family caregivers (controls), matched for sex and aligned closely by age (±5 years), were enrolled. Clinical information about the ALS patients are reported in Table 1. The mean age at enrollment was 59 (range 40–79) and 70% of patients were male. Of the recruited patients 37% met the El Escorial criteria of definite ALS (EEC-R0), 32% were clinical probable (EEC-R1), 5% were probable-laboratory-supported (EEC-R2), and 26% were possible ALS (EEC-R1). Ninety percent of the patients had spinal onset and 10% had bulbar onset disease. Five percent had a Bulbar phenotype, 47% Classic, 21% Flail arm/leg and 26% had a UMNp phenotype. Forty two percent of patients has a fast progression rate, 42% slow and 16% medium. The average ALSFRS-R score at sampling was 32 (SD ± 7.45).

### 3.2. Serum Cytokine Profile

**Figure 1:**
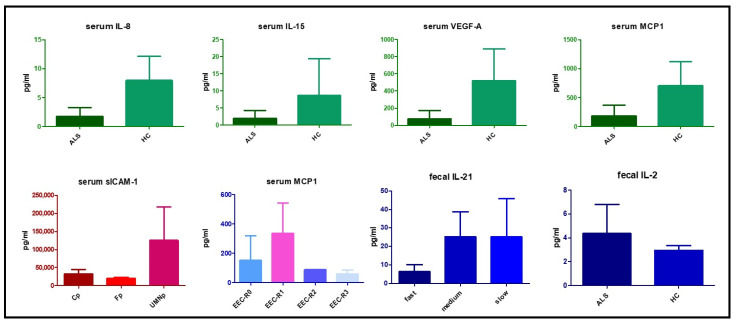
Serum and fecal cytokines with a significant different distribution among patient subgroups and controls. ALS= amyotrophic lateral sclerosis. HC = healthy controls. EEC = EL Escorial Criteria; EEC-R0 = definite; EEC-R1 = clinically probable; EEC-R2 = probable-laboratory-supported; EEC-R3 = possible. Bp = Bulbar phenotype; Cp = Classic phenotype; Fp = Flail arm/leg phenotype; UMNp = Upper Motor Neuron predominant phenotypes.

We observed that GM-CSF, G-CSF, IL-5, IL-6, IL-9, IL-12(p70), IL-13, IFN-α, IL-23, IL-27 were below the quantification limit in all serum samples (patients and controls). Significant differences in some cytokine levels were observed among patient subgroups and controls (Figure 1): IL-8 (1.79 ± 1.49 vs. 7.75 ± 3.97 pg/mL; *p* = 0002), IL-15 (1.91 ± 2.31 vs. 8.57 ± 10.75 pg/mL; *p* < 0.0001), MCP-1 (185.08 ± 186.26 vs. 677.34 ± 392.10 pg/mL; *p* = 0.002) and VEGF-A (75.74 ± 97.52 vs. 518.9 ± 372.0 pg/mL; *p* < 0.0001) levels were significantly lower in patients than controls, while others did not show a different distribution among groups. Patients with different ALS progression rates did not display different serum cytokine distributions. Differences were observed in clinical phenotype subgroups: IL-2 was only detected in the single bulbar patient phenotype; sICAM-1 was significantly higher in UMN patients (125,483.39 ± 92,894.77 pg/mL) than other subgroups, including flail arm/leg (20,206.29 ± 3296.96 pg/mL) and classic (22,623.38 ± 11,988.63 pg/mL) phenotypes (*p* = 0.044). MCP-1 was differentially concentrated in ALS patients according to the El Escorial clinical criteria, showing higher values in patients with a clinical probable score (337.25 ± 207.10 pg/mL) than patients with a definite (153.09 ± 167.63 pg/mL) or possible (60.24 ± 24.76 pg/mL) (*p* = 0.016) score. Finally, IP-10 levels were positively correlated with survival (*p* = 0.039). Cytokine levels are reported in Supplementary Table S2.

### 3.3. Fecal Cytokine Profile

IFN-γ, IL-10, TNF-α, IL-5, IL-12p70, IL-13, GM-CSF, G-CSF, IL-15, VEGF-A, IFN-α, IL-9, IL-23, IL-8, IL-17A and IP-10 were under the LLOQ in all fecal extracts (ALS and controls). IL-2 was higher in ALS fecal samples compared to controls (4.36 ± 2.42 vs. 2.96 ± 0.40; *p* = 0.039) and IL-1β was also higher in ALS patients (6.20 ± 13.55 vs. 2.10 ± 2.68 pg/mL; *p* = 0.536), but not significantly. The other cytokines (MIP-1α, IL-27, IL-6, MCP-1, P-selectin, IL-1α, IL-18, IL-21, IL-22, sICAM1, E selectin) were detected in only a few ALS patients but not in control fecal samples. Regarding ALS patients, IL-21 was lower in patients with a fast progression compared to slow or medium progressors (6.3 ± 3.83 pg/mL vs. 25.1 ± 20.7 vs. 25.3 ± 13.48, *p* = 0.029) (Figure 1). Fecal cytokines levels are reported in Supplementary Table S3.

### 3.4. Profile of Fecal SCFAs

The fecal SCFA concentrations (µmol/g) were not significantly different between ALS patients and controls, or between patients with different clinical characteristics. The concentrations of SCFAs in patients and controls are reported in Table 2.

### 3.5. Definition of the Gut Microbiota Composition 

**Figure 2:**
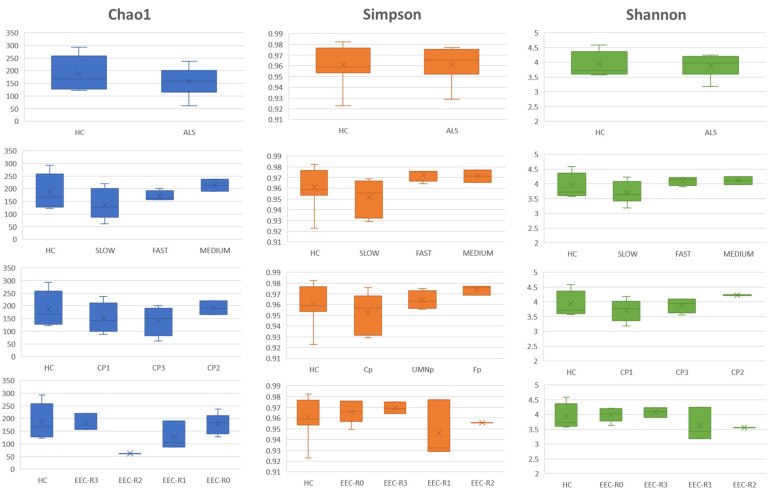
Boxplots displaying the Chao1, Simpson and Shannon alpha-diversity index of fecal samples among patient subgroups and controls. ALS = amyotrophic lateral sclerosis. HC = healthy controls. EEC = EL Escorial Criteria; EEC-R0 = definite; EEC-R1 = clinically probable; EEC-R2 = probable-laboratory supported; EEC-R3 = possible. Bp = Bulbar phenotype; Cp = Classic phenotype; Fp = Flail arm/leg phenotype; UMNp = Upper Motor Neuron predominant phenotypes.

To determine if ALS was associated with a change in the microbiota composition and structure, fecal samples from ALS patients and controls were subjected to 16S targeted metagenomic sequencing. Analysis of alpha diversity in samples from controls and ALS patients did not reveal any significant difference according to the Chao1, Simpson and Shannon diversity indices (Figure 2). Nevertheless, although not statistically significant, specific trends were identified in some patient categories: patients characterized by a slow progressing ALS showed a lower microbial diversity (Chao1, Simpson, Shannon) than the other progression phenotypes, as well as patients with classical ALS (CP) compared to patients classified with different clinical phenotypes.

**Figure 3:**
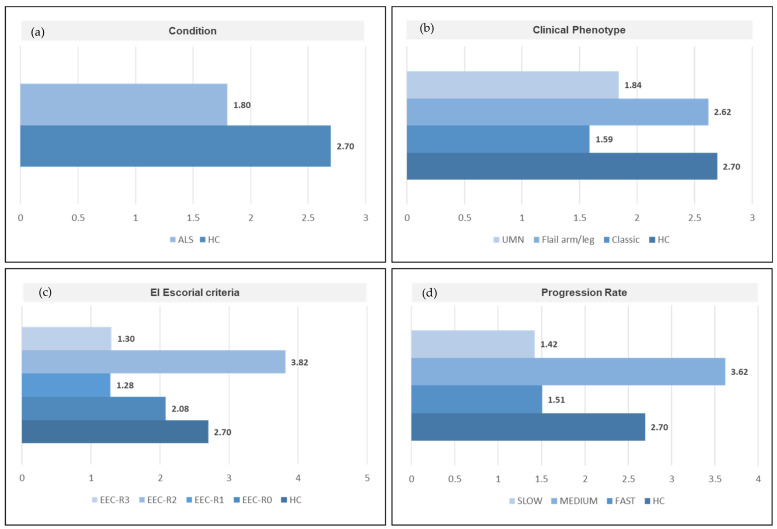
Differences in the Firmicutes to Bacteroidetes ratio according to (**a**) condition (ALS patients vs. healthy controls), (**b**) ALS clinical phenotype; (**c**) El Escorial Criteria; (**d**) rate of ALS progression. ALS= amyotrophic lateral sclerosis. HC= healthy controls. EEC = EL Escorial Criteria; EEC-R0 = definite; EEC-R1 = clinically probable; EEC-R2 = probable-laboratory-supported; EEC-R3 = possible. Bp = Bulbar phenotype; Cp = Classic phenotype; Fp = Flail arm/leg phenotype; UMNp = Upper Motor Neuron predominant phenotypes.

Evaluation of beta-diversity through the Bray–Curtis and UniFrac (weighted, unweighted) metrics did not reveal a marked sample separation according to the main study group, including healthy controls and ALS patients (PERMANOVA *p* > 0.05), nor according to the clinical phenotypes or El Escorial classification criteria subgroups of ALS patients (PERMANOVA *p* > 0.05) (Figures S1–S3). Conversely, samples from patients with slow and fast progression rates showed a trend in clustering away from each other according to the Bray–Curtis and the Weighted UniFrac metrics, although without statistical significance (PERMANOVA *p* = 0.061 and *p* = 0.588, respectively). Analysis of the taxonomic composition revealed that Firmicutes and Bacteroides were the most represented phyla in all samples (Figure S4), and that their ratio (F/B) showed a wide variation within the study groups and ALS subgroups (Figure 3). Overall, control samples showed a higher F/B than samples from ALS patients, although with some differences. Indeed, ALS patients diagnosed with a medium progression rate showed higher F/B ratios compared to both controls and other ALS patients within the corresponding subgroups. No statistically significant differences between those F/B ratios were identified, nor significant correlation between F/B ratios and the rate of ALS progression or the survival (days) from symptom onset.

**Figure 4:**
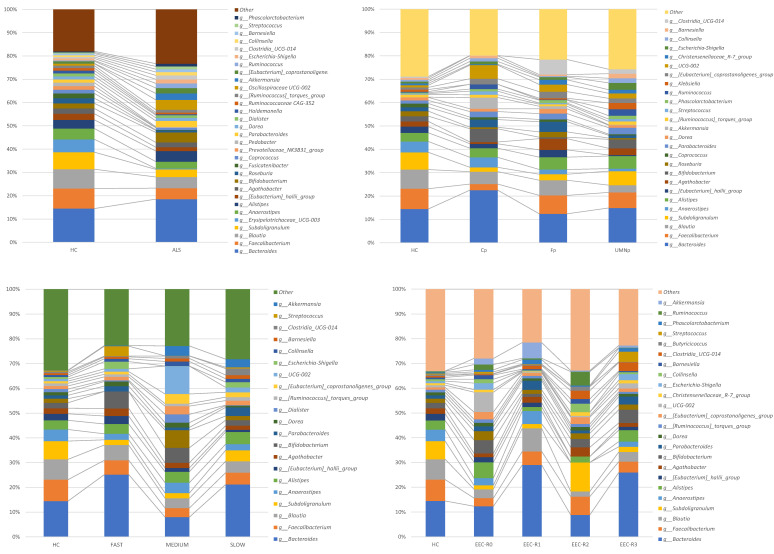
Stacked bar plots displaying the average relative abundance of bacterial amplicon sequence variants (ASVs) identified at the family taxonomic level in the gut microbiota of ALS patient subgroups and healthy controls. ALS= amyotrophic lateral sclerosis. HC= healthy controls. EEC= EL Escorial Criteria; EEC-R0= definite; EEC-R1 = clinically probable; EEC-R2 = probable-laboratory-supported; EEC-R3 = possible. Bp = Bulbar phenotype; Cp = Classic phenotype; Fp = Flail arm/leg phenotype; UMNp = Upper Motor Neuron predominant phenotypes.

At a lower taxonomic level, the *Lachnospiraceae*, *Ruminococcaceae* and *Bacteroidaceae* were the most represented bacterial families in all study groups and subgroups, collectively accounting for a relative abundance of more than 40% (Figure 4). 

**Figure 5:**
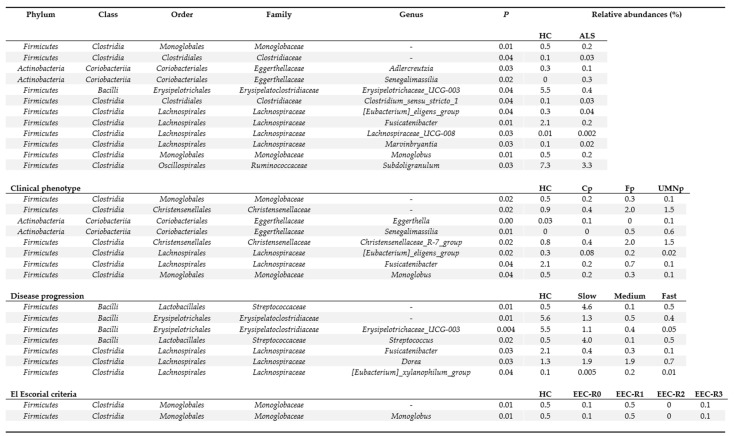
Significant differentially abundant taxa among ALS patients and controls. ALS = amyotrophic lateral sclerosis. HC = healthy controls. EEC = EL Escorial Criteria; EEC-R0 = definite; EEC-R1 = clinically probable; EEC-R2 = probable-laboratory-supported; EEC-R3 = possible. Bp = Bulbar phenotype; Cp = Classic phenotype; Fp = Flail arm/leg phenotype; UMNp = Upper Motor Neuron predominant phenotypes.

Overall, differential abundance analyses revealed that most of the significant differences were associated with bacterial taxa belonging to the *Lachnospiraceae* family (Figure 5). At genus level, control patients showed enrichment of *Erysipelotrichaceae_UCG-003 (Erysipelatoclostridiaceae fam.)*, *Fusicatenibacter (Lachnospiraceae fam.)*, and *Subdoligranulum (Ruminococcaceae fam.*) compared to ALS patients (Figure S5). Interestingly, the relative abundance of Erysipelotrichaceae_UCG-003 *(Erysipelatoclostridiaceae fam.)* and *Fusicatenibacter (Lachnospiraceae fam.)* showed a significant progressively decreasing trend according to the rate of disease progression in ALS patients compared to controls. Within the same subgroup of ALS patients, a marked increase of *Streptococcus* was identified in samples from patients with a slow progression (Figure 5).

**Figure 6:**
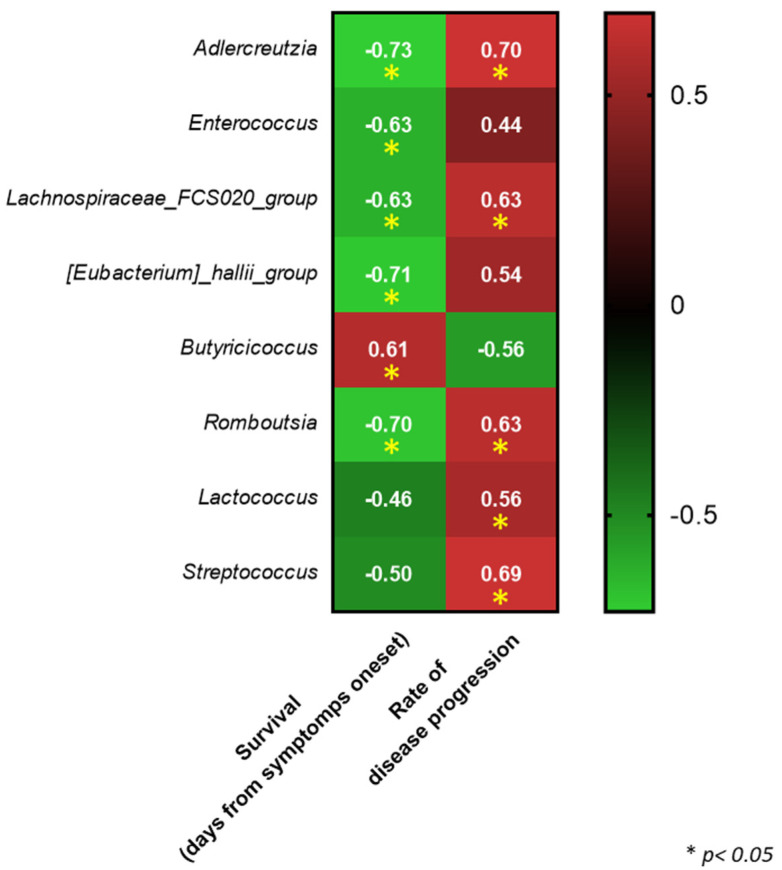
Heatmap of significant correlations (Spearman) identified between taxa relative abundances and disease progression or ALS patients’ survival (days) from symptom onset.

Furthermore, significant negative and positive correlations were respectively identified between the abundance of some genera and disease progression and the patients’ survival (days) from symptom onset (Figure 6). Additional correlation analyses were performed between taxa relative abundances, at a genus level, and the fecal content of SCFAs.

**Figure 7:**
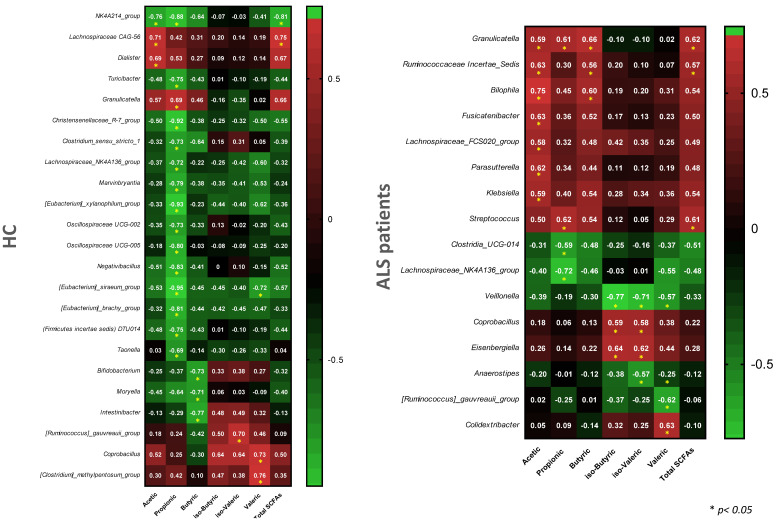
Heatmap of significant correlations (Spearman) identified between taxa relative abundances and levels of fecal SCFAs in ALS patients and healthy controls.

Results revealed that controls and ALS patients were characterized by different correlation profiles, with the controls including many genera negatively correlated with Propionic acid, and the ALS patients including some genera positively correlated with Acetic acid (Figure 6). Few genera simultaneously showed significant correlations in both controls and ALS patient study groups, including the *Granulicatella*, *Lachnospiraceae*_NK4A136_group, *Coprobacillus* and *Ruminococcus gauvreauii* group, among which the *Granulicatella* and *Lachnospiraceae*_NK4A136_groups showed a consistent positive and negative correlation, respectively, with Propionic acid (Figure 7).

## 4. Discussion

Increasing studies, even if sometimes contrasting, support the role of the microbiota-immunity axis as new player in the pathogenesis of neurodegenerative diseases, including ALS. The microbiota may influence the CNS and neuronal health in different ways; directly, by producing neuroactive metabolites or toxins, and indirectly, by modulating the immune system, as recently reviewed in [34].

In this study, we evaluated the microbiota composition and the inflammatory response of ALS patients compared to cohabiting controls. In addition, we explored their role as potential biomarkers of clinical heterogeneity in ALS. First, we analyzed the systemic and intestinal inflammatory status, measuring a panel of 30 inflammatory-correlated cytokines in serum and fecal samples. Compared to controls, ALS patients showed a different serum profile, characterized by lower amounts of specific cytokines (IL-15, IL-8, MCP-1 and VEGF-A).

The dysregulation of the inflammatory response has been largely documented in ALS by the alteration of several pro- and anti-inflammatory cytokines, both in the blood and cerebrospinal fluid (CSF) of patients compared to controls and/or patients with other non-inflammatory neurological diseases [35,36,37,38,39]. Moreover, numerous interleukins have been correlated with faster or slower progression [39,40]. Although many studies have documented an upregulation of serum inflammatory biomarkers in ALS, we did not find any over-expressed cytokines in the serum of patients compared to controls. For example, we observed a lower amount of the two chemokines IL-8 and MCP-1. Previously, Ehrhart and colleagues also documented a decrease in some circulating cytokines (IL-5, IL-2, GM-CSF) compared to healthy subjects. However, literature data also report elevated serum and CSF levels of IL-8 and MCP-1 in ALS [41,42]. 

In the central nervous system, IL-8 is produced by glial cells where it regulates the recruitment and activation of polymorphonuclear cells, with the consequent release of cytokines and tissue damage mediators [43,44], those playing a role in mediating neuronal damage in several neurological disorders [42]. However, a reduction of serum and CSF levels of IL-8 has been demonstrated in other neurological disorders, such as Alzheimer disease [45,46]. Because IL-8 has a role in promoting neuronal survival [47], its decrease could be associated with declined reparative mechanisms in the central nervous system. 

The monocyte chemoattractant protein-1, MCP-1, is an important mediator of neuroinflammation and microglial activation [48]. We found lower levels of MCP-1 in ALS patients’ serum, in accordance with another recent study by Modgil’s group [49]. They, besides studying peripheral neuroinflammatory markers, have also explored the expression of proteins involved in angiogenesis (including VEGF-A) and proteinopathy in Indian ALS patients. The VEGF-A is usually reported to be higher in the serum and CSF of ALS patients compared to controls, and its levels seems to augment as the disease progresses [50]. In agreement with Modgil and other groups, we reported lower serum levels of VEGF in patients compared to controls [51,52]. Interestingly, different cytokine profiles have been identified among ALS subgroups, since MCP-1 was higher in patients with a clinically probable EL Escorial diagnostic category, and sICAM was higher in patients with a UMNp clinical phenotype, suggesting their potential relationship with disease progression or the kind of MN involvement. Moreover, we also observed a positive correlation between the IP-10 level and patients’ survival. 

Furthermore, for the IL-2 role in ALS there are contrasting studies. IL-2 is an immunoregulatory cytokine secreted by T cells and modulates T helper subset differentiation. Some studies documented an increased IL-2 level in ALS and its association with a poorer survival [37,39]; while others observed a significant IL-2 decrease in patients’ sera, especially at a more pronounced disease stage [38]. Interestingly, reduced IL-2 expression can alter not only the proliferation of pro-inflammatory Th1/Th17 subsets (promoting ALS progression) but also the proliferation of protective anti-inflammatory Treg/Th2 cells [38]. However, we did not observe differences in serum IL-2 among patients and controls, even though we found consistently higher IL-2 levels in fecal samples of ALS patients compared to controls.

Regarding the fecal inflammatory-correlated biomarkers, our results have documented, for the first time, an intestinal inflammatory tone in ALS patients. Indeed, a large number of cytokines were measurable only in ALS fecal samples (MIP-1α, IL-27, IL-6, MCP-1, P-selectin, IL-1α, IL-18, IL-21, IL-22, sICAM1, E selectin) and, as previously mentioned, IL-2 was significantly higher in patients than controls. The fecal cytokine profile of patients did not show differences according to the clinical phenotype and diagnostic criteria but, interestingly, the IL-21 expression was higher in patients with slow versus high progression rates, suggesting its potential role as a prognostic biomarker. 

Regarding the fecal microbiota composition, ALS patients and healthy cohabiting controls showed a similar phenotypical and functional structure, with minor differences. Indeed, as demonstrated by the α-diversity analysis, samples belonging to the two groups displayed a comparable microbial diversity, richness and evenness. Likewise, the between-sample diversity in the microbial composition and structure did not differ significantly, as demonstrated by PCoA calculated with the Bray–Curtis, weighted and unweighted UniFrac β-diversity metrics. Conversely, the taxonomic compositional analysis showed a lower Firmicutes/Bacteroides ratio (a dysbiotic indicator) in patients compared to controls. Findings about the F/B ratio in ALS are quite controversial, since while some studies have reported a reduced ratio [18,19], others have reported the opposite [17], or no change [21]. In the latter case, however, a higher F/B ratio has been associated with an increased risk of death in ALS patients [21]. Furthermore, at a lower taxonomic level, ten specific genera were found to be significantly less abundant in ALS patients; these taxa, most belonging to the *Lachnospiraceae* and *Ruminococcaceae* family, included many known butyrate-producing organisms. Overall, our results are consistent with previous findings, since other researchers did not find substantial taxonomic or metagenomics differences in patients vs. controls [20,21], apart from the different abundances of specific genera (in particular *Ruminococcus*) [17,18,19]. Interestingly, we observed a marked reduction of *Subdoligranulum*, an anaerobic, non-spore-forming, butyrate-producing, Gram-negative bacterium, in ALS patients compared to controls. The reduction of butyrate-producing bacteria has been previously documented in ALS [53,54] as in other immune-mediated deregulatory conditions, and butyrate supplementation appeared to ameliorate the clinical features of ALS and the immunological abnormalities found in the ALS mice model [55]. In addition, several findings suggest a beneficial role of *Subdoligranulum* on host metabolism, hypothesizing its role as probiotic candidate to improve the host metabolic health [56,57]. A depletion of *Subdoligranulum* (*Clostridium* cluster) and butyrate has been recently described in Behçet’s syndrome, a neutrophilic vasculitis with gastrointestinal involvement [58]. Butyrate exerts an important role for the maintenance of host immune homeostasis, prompting the generation of extrathymic Tregs, and inhibiting local pro-inflammatory cytokines [59,60,61], and a butyrate-rich diet can improve blood redox status and fibrin degradation, which is impaired by a neutrophil-dependent mechanism (via ROS) in Behçet’s syndrome [62]. In ALS, an unbalance of the *Firmicutes*/*Bacteroides* ratio and the alteration of butyrate levels may affect ALS pathogenesis by affecting Treg/Th17 balances [63]. Notably, the imbalance of oxidative mechanisms represents another pathogenic mechanism in ALS [1] and NOX2 activity was downregulated in peripheral neutrophils of ALS patients [64]. Nevertheless, in our study, the profile of SCFAs, including butyrate levels, was similar in patients and controls. On the other hand, ALS patients displayed different correlation patterns, even opposite, between distinct bacterial genera and specific SCFAs, in particular propionic acid, compared to controls. Interestingly, propionic acid may have an important role for nervous system health, since its excess seems to have several neurotoxic effects, including neuroinflammation, mitochondrial dysfunction and glutamate excitotoxicity [65,66,67].

Some differences in the microbiota composition were also identified among patients with a distinct clinical phenotype. Indeed, patients with a classical phenotype showed a lower microbial diversity compared to others, and UMNp patients, that usually have a milder disease progression, had higher amounts (although not significant) of fecal SCFAs. Regarding the disease progression rate, patients with a slow progression stand out from the others for their reduced α-diversity, a lower F/B ratio and the higher abundance of bacteria belonging to the *Streptococcaceae* family (in particular, the *Streptococcus* genus, that progressively decreases with the progression rate increment). Furthermore, we observed various significant negative and positive correlations between some taxa and ALS progression or patients’ survival. Further investigations are needed to assess the role of such taxa as potential microbial biomarkers in ALS patients.

Overall, our study confirmed the dysregulation of the inflammatory status of ALS patients, both systemically and at the intestinal level, together with an alteration of the intestinal bacteria community structure. In particular, the characterization of the gut microbiota revealed differences between patients and cohabiting peoples, marked by a reduction of butyrate-producing taxa that, according to a growing body of evidence, could promote an inflammatory tone. Nevertheless, other researchers have documented a more consistent difference in the taxonomic and metagenomics microbiota composition of ALS patients compared to healthy subjects that we did not observe [53,68,69].

There can be many explanations for these discrepancies, such as the limited patient numbers, suboptimal study designs, differences in sequencing methods, and the presence of different confounding factors (diet, secondary disease effects, dysphagia, etc.) or the large heterogeneity (genetic, phenotypic and pathophysiologic) that characterizes the ALS disease [16]. Of note, some differences in the inflammatory/microbial profile did not emerge in the comparison between patients and controls, but they were visible only when comparing specific subgroups of patients. Although in a small number of patients, indeed, we evaluated the existence of differences in the inflammatory and microbial profile primarily depending on the clinical and laboratory patient subgroups, confirming a high interindividual variability among patients regarding those aspects. The well-established heterogeneity of ALS negatively affects the planning and interpretation of clinical trials, nullifying the effort to understand the disease mechanisms and to find favourable treatments. Therefore, successfully stratifying patients into clinically meaningful subgroups represents an important challenge in ALS management. Based on our results and other literature evidence, we believe that taking care of the genetics and biology of the “holobiont” (the host and its microbiota) and its massive interactions, and following a holistic approach, will be of great value to clarify the fine biological mechanisms underlying ALS and to develop new effective and personalized treatments.

## Figures and Tables

**Table 1:** Summary of ALS patients’ clinical characteristics.

Characteristics	ALS Patients
Sex, %male	70%
Age at enrollment, mean (SD)	59 (12)
Height (cm), mean (SD)	173 (7.6)
Weight (kg), mean (SD)	80 (13.5)
BMI, mean (SD)	26.8 (4.3)
ALSFRS-R at sampling, mean (SD)	32 (7.4)
Clinical Phenotype, n (%)	Bulbar, 1 (5)
	Classic, 9 (47)
	Flail arm/leg, 4 (21)
	UMNp, 5 (26)
El Escorial, n (%)	Definite, 7 (37)
	Clinical probable, 6 (32)
	Laboratory-supported, 1 (5)
	Fast, 8 (42)
Progression rate, n (%)	Slow, 8 (42)
	Medium, 3 (16)
	Fast, 8 (42)
Survival (days from symptom onset, mean (SD))	1929 (1374.7)
Site onset, n (%)	Bulbar, 2 (10)
	Spinal, 17 (90)

**Table 2:** Mean ± standard deviation of each short chain fatty acid concentration in patients and healthy controls. ALS= amyotrophic lateral sclerosis. HC = healthy controls. EEC = EL Escorial Criteria; EEC-R0 = definite; EEC-R1 = clinically probable; EEC-R2 = probable-laboratory-supported; EEC-R3 = possible. Bp = Bulbar phenotype; Cp = Classic phenotype; Fp = Flail arm/leg phenotype; UMNp = Upper Motor Neuron predominant phenotypes.

Condition		Acetic Acid (umol/g)	Propionic Acid (umol/g)	Butyric Acid (umol/g)	Iso-Butyric Acid (umol/g)	Iso-Valeric Acid (umol/g)	Valeric Acid (umol/g)	Total (umol/g)
HC		27.05 ± 10.59	8.79 ± 3.48	7.28 ± 3.99	1.43 ± 0.81	1.15 ± 0.89	1.28 ± 0.40	54.22 ± 19.88
ALS (all patients)		22.89 ± 13.9	8.05 ± 5.03	8.80± 6.92	1.49 ± 1.28	0.95 ± 1.23	1.36 ± 1.21	52.93 ± 34.23
ALS Subgroups								
El Escorial Criteria	EEC-R0	17.91 ± 11.06	5.72 ± 4.78	6.79 ± 6.19	1.05 ± 1.48	0.95 ± 1.31	1.07 ± 1.28	37.73 ± 31.80
	EEC-R1	15.76 ± 12.06	7.83 ± 4.22	4.84 ± 6.42	1.03 ± 1.30	0.85 ± 1.49	1.72 ± 1.46	35.62 ± 30.28
	EEC-R2	2.80	1.54	0.69	0.51	0.63	0.30	7.10
	EEC-R3	27.22 ± 17.27	5.75 ± 6.67	7.81 ± 8.67	1.76 ± 1.23	1.61 ± 1.09	1.13 ± 0.88	53.18 ± 41.38
Clinical phenotype	Cp	16.90 ± 16.54	7.83 ± 4.92	6.05 ± 7.38	0.65 ± 1.19	0.54 ± 0.98	1.25 ± 1.19	37.07 ± 37.51
	Bp	17.68	5.72	4.54	0.35	0.28	1.07	34.08
	Fp	22.81 ± 5.27	5.25 ± 0.71	6.38 ± 1.99	1.76 ± 0.50	1.61 ± 0.49	1.13 ± 0.19	45.74 ± 10.93
	UMN-p	20.00 ± 15.20	12.17 ± 6.83	15.38 ± 8.49	3.07 ± 1.53	2.70 ± 1.56	2.68 ± 1.67	75.16 ± 42.11
Progression rate	Slow	19.50 ± 12.37	6.07 ± 4.62	5.85 ± 6.38	1.40 ± 1.12	1.28 ± 1.04	1.26 ± 1.11	41.08 ± 31.99
	Medium	17.91 ± 13.28	7.48 ± 3.41	6.79 ± 5.34	1.35 ± 0.46	1.12 ± 0.44	1.67 ± 0,78	37.73 ± 27.31
	Fast	18.84 ± 17.09	8.78 ± 6.28	9.09 ± 8.40	0.69 ± 1.69	0.56 ± 1.64	1.05 ± 1.54	54.62 ± 41.77

## Data Availability

The 16S rRNA sequence data have been deposited in the NCBI Sequence Read Archive (SRA) database (https://www.ncbi.nlm.nih.gov/bioproject/, accessed on 16 April 2021) under the BioProject accession number PRJNA736213.

## Supplementary Materials

The following are available online at https://www.mdpi.com/article/10.3390/biomedicines9070753/s1, Table S1: Lower Limit of Quantification (LLOQ, pg/mL) for each evaluated cytokine. Table S2. Mean ± standard deviation of serum cytokines in patients and healthy controls. Table S3. Mean ± standard deviation of fecal cytokines in patients and healthy controls. Figure S1: Principal coordinates analysis (PCoA) according to the Bray–Curtis beta-diversity metric. Figure S2: Principal coordinates analysis (PCoA) according to the Unweighted UniFrac beta-diversity metric. Figure S3: Principal coordinates analysis (PCoA) according to the Weighted UniFrac beta-diversity metric. Figure S4: Stacked bar plots displaying the average relative abundance of bacterial amplicon sequence variants (ASVs) identified at the phylum taxonomic level in the gut microbiota of ALS patient subgroups and healthy controls. Figure S5: Stacked bar plots displaying the average relative abundance of bacterial amplicon sequence variants (ASVs) identified at the genus taxonomic level in the gut microbiota of ALS patient subgroups and healthy controls.
