# Supplementary material for: The Gut Microbiota-Immunity Axis in ALS: A Role in Deciphering Disease Heterogeneity?
Source: Biomedicines. 2021 Jun 29;9(7):753. doi: 10.3390/biomedicines9070753 (PMC8301418; doi:10.3390/biomedicines9070753)
Supplement: Supplementary file 1 [file biomedicines-09-00753-s001.zip › biomedicines-1279458-supplementary/biomedicines-1279458-Supplementary-done.pdf]

biomedicines

Article

The Gut Microbiota-Immunity Axis in ALS: A Role in Deciphering Disease Heterogeneity?

Elena Niccolai [1,†], Vincenzo Di Pilato [2,†], Giulia Nannini [1], Simone Baldi [1], Edda Russo [1], Elisabetta Zucchi [3], Ilaria Martinelli [4], Marta Menicatti [5], Gianluca Bartolucci [5], Jessica Mandrioli [3,4,*] and Amedeo Amedei [1,*]

[1] Department of Clinical and Experimental Medicine; University of Florence, 50134 Florence, Italy; elena.niccolai@unifi.it (E.N.); simone.baldi@unifi.it (S.B.); giulia.nannini@unifi.it (G.N.); edda.russo@unifi.it (E.R.).
[2] Department of Surgical Sciences and Integrated Diagnostics (DISC), University of Genoa, 16132, Genoa, Italy; vincenzo.dipilato@unige.it
[3] Department of Biomedical, Metabolic and Neural Sciences, University of Modena and Reggio Emilia, 41125 Modena, Italy; elibettizucchi@gmail.com
[4] Neurology Unit, Department of Neuroscience, Azienda Ospedaliero Universitaria di Modena, 41125 Modena, Italy; martinelli.ilaria88@gmail.com.
[5] Department of Neurosciences, Psychology, Drug Research and Child Health Section of Pharmaceutical and Nutraceutical Sciences, University of Florence, 50139 Florence, Italy; gianluca.bartolucci@unifi.it (G.B.); marta.menicatti@unifi.it (M.M.)
* Correspondence: amedeo.amedei@unfi.it; Tel.: +39 0552758330 (A.A.) and mandrioli.jessica@aou.mo.it; +39 0593961700 (J.M.)
† These authors contributed equally to this work

Table S1. Low Limit of Quantification (LLOQ, pg/ml) for each evaluated cytokine.

Cytokine	LLOQ (pg/ml)
G-CSF	13.00
IFN-γ	7.47
IL-1β	2.05
IL-2	4.27
IL-4	11.00
IL-6	8.15
IL-8	2.17
IL-10	2.25
IL-15	3.05
IL-17A	1.81
MCP-1	4.76
MIP-1α	1.56
TNFα	6.52
VEGF-A	1.19
IL-27	24.00
IL-5	6.42
IP-10	2.15
IL-12p70	6.40
IL-13	2.15
GM-CSF	13.00
IFN-α	0.55
IL-9	7.69
P-selectin	1233.00
IL-1α	0.54
IL-23	14.00

IL-18	6.84
IL-21	7.79
sICAM1	212.00
IL-22	26
E-selectin	250.00

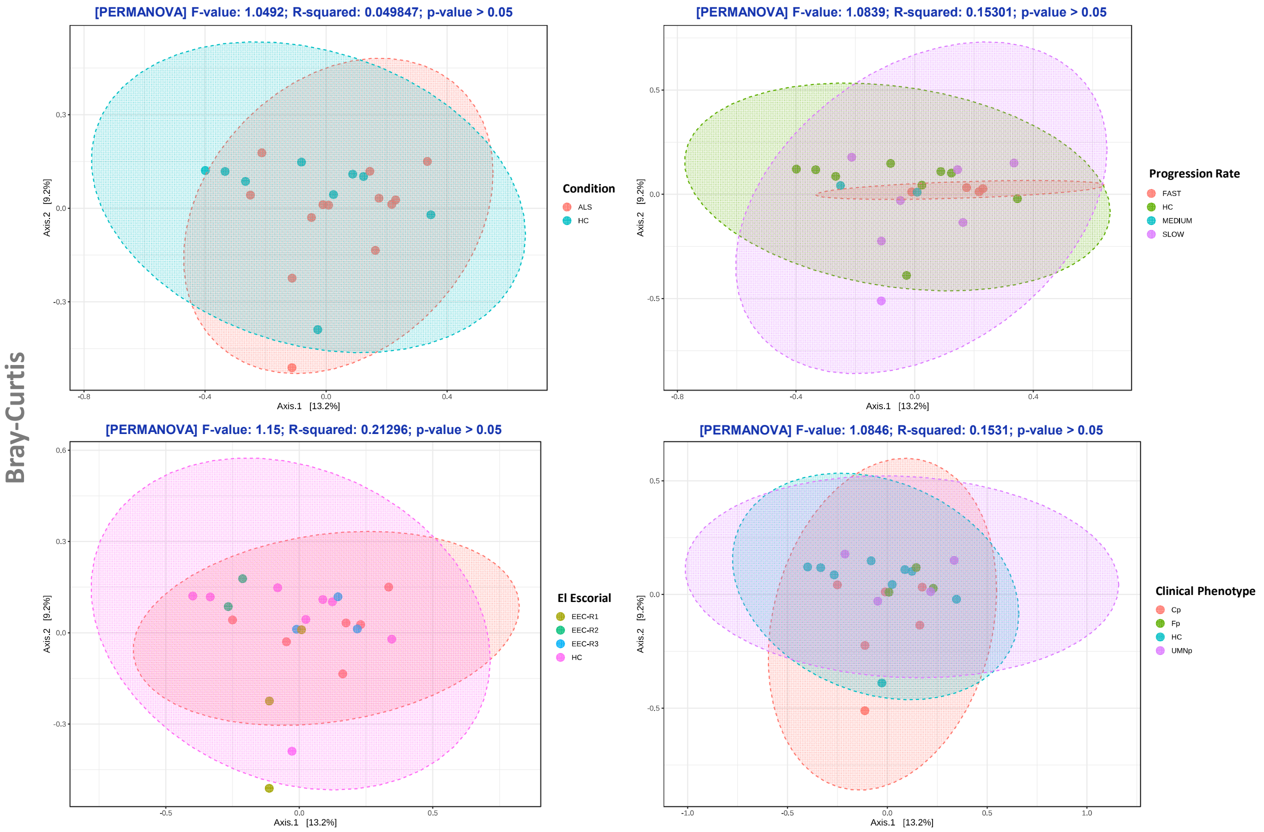

Figure S1. Principal coordinates analysis (PCoA) according to the Bray-Curtis beta-diversity metric. Results of the permutational multivariate analysis of variance (PERMANOVA) are also shown. Subjects are colored according to the group: a) condition (ALS patients vs healthy controls), b) rate of ALS progression c) El Escorial Criteria d) clinical phenotype. ALS= amyotrophic lateral sclerosis. EEC= EL Escorial Criteria; EEC-R0= definite; EEC-R1=clinically probable; EEC-R2=probable-laboratory supported, EEC-R3= possible. Bp= bulbar phenotype; Cp= Classic phenotype; Fp= Flail arm/leg phenotype; UMNp= Upper Motor Neuron predominant phenotypes.

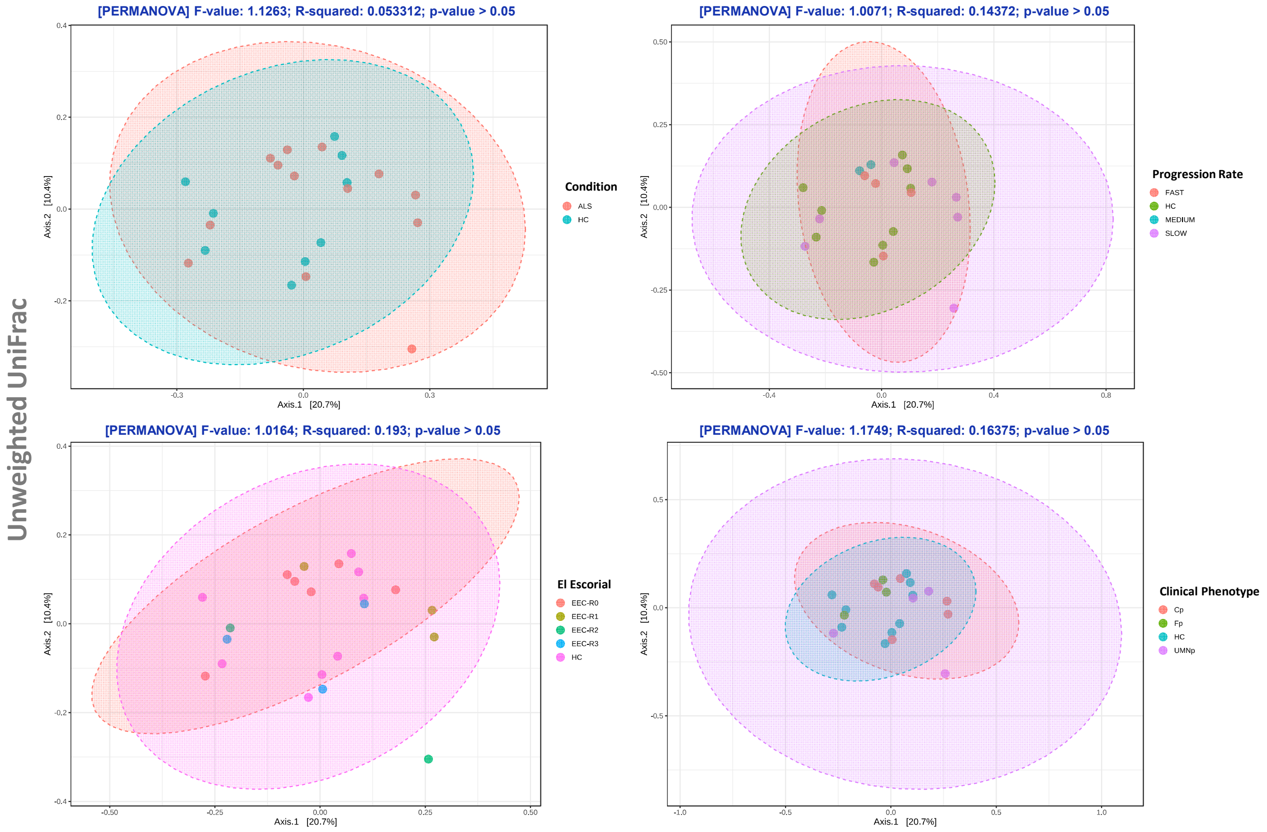

Figure S2. Principal coordinates analysis (PCoA) according to the Unweighted UniFrac beta-diversity metric. Results of the permutational multivariate analysis of variance (PERMANOVA) are also shown. Subjects are colored according to the group: a) condition (ALS patients vs healthy controls), b) rate of ALS progression c) El Escorial Criteria d) clinical phenotype. ALS= amyotrophic lateral sclerosis. EEC= EL Escorial Criteria; EEC-R0= definite; EEC-R1= possible, EEC-R2=clinically probable; EEC-R3=probable-laboratory supported. Cp= Classic phenotype; Fp= Flail arm/leg phenotype; UMNp= Upper Motor Neuron predominant phenotypes.

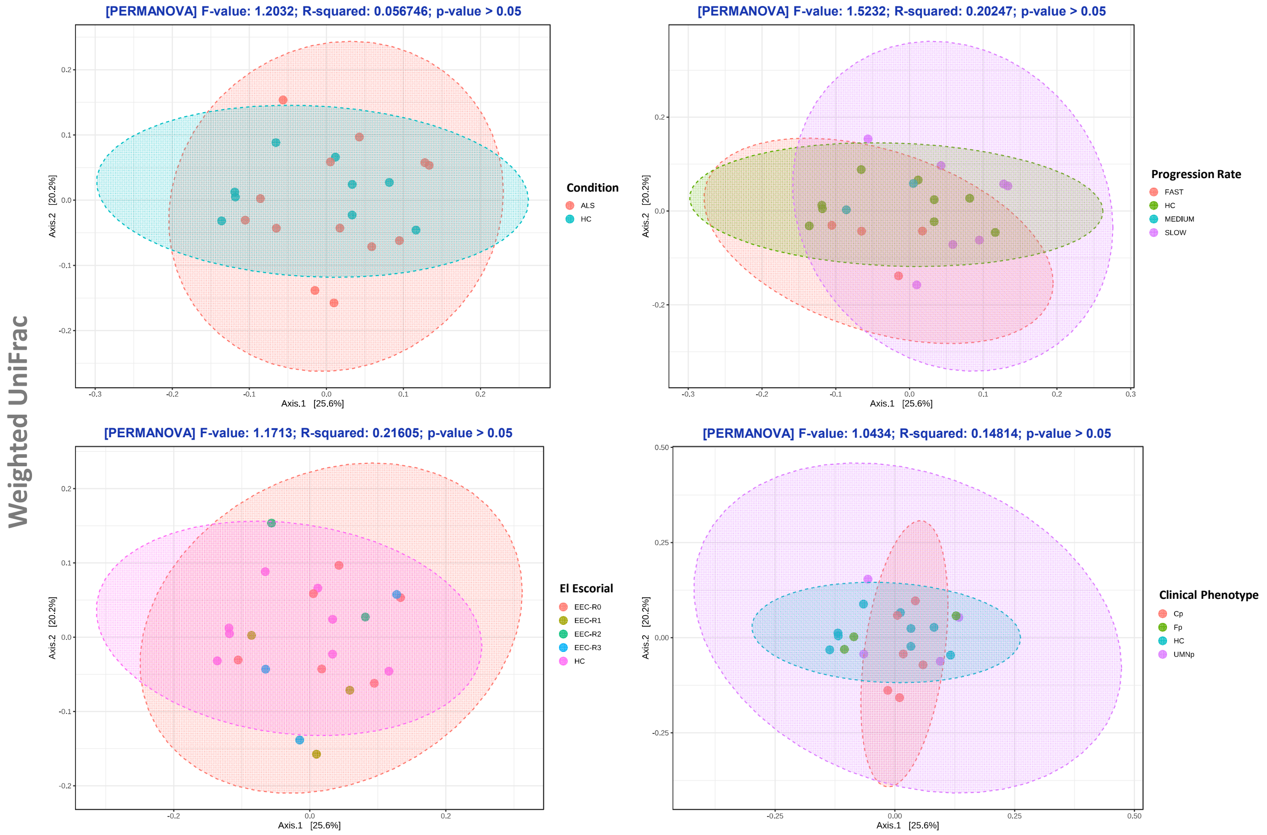

Figure S3. Principal coordinates analysis (PCoA) according to the Weighted UniFrac beta-diversity metric. Results of the permutational multivariate analysis of variance (PERMANOVA) are also shown. Subjects are colored according to the group: a) condition (ALS patients vs healthy controls), b) rate of ALS progression c) El Escorial Criteria d) clinical phenotype. ALS= amyotrophic lateral sclerosis. EEC= EL Escorial Criteria; EEC-R0= definite; EEC-R1= possible, EEC-R2=clinically probable; EEC-R3=probable-laboratory supported. Bp= bulbar phenotype; Cp= Classic phenotype; Fp= Flail arm/leg phenotype; UMNp= Upper Motor Neuron predominant phenotypes.

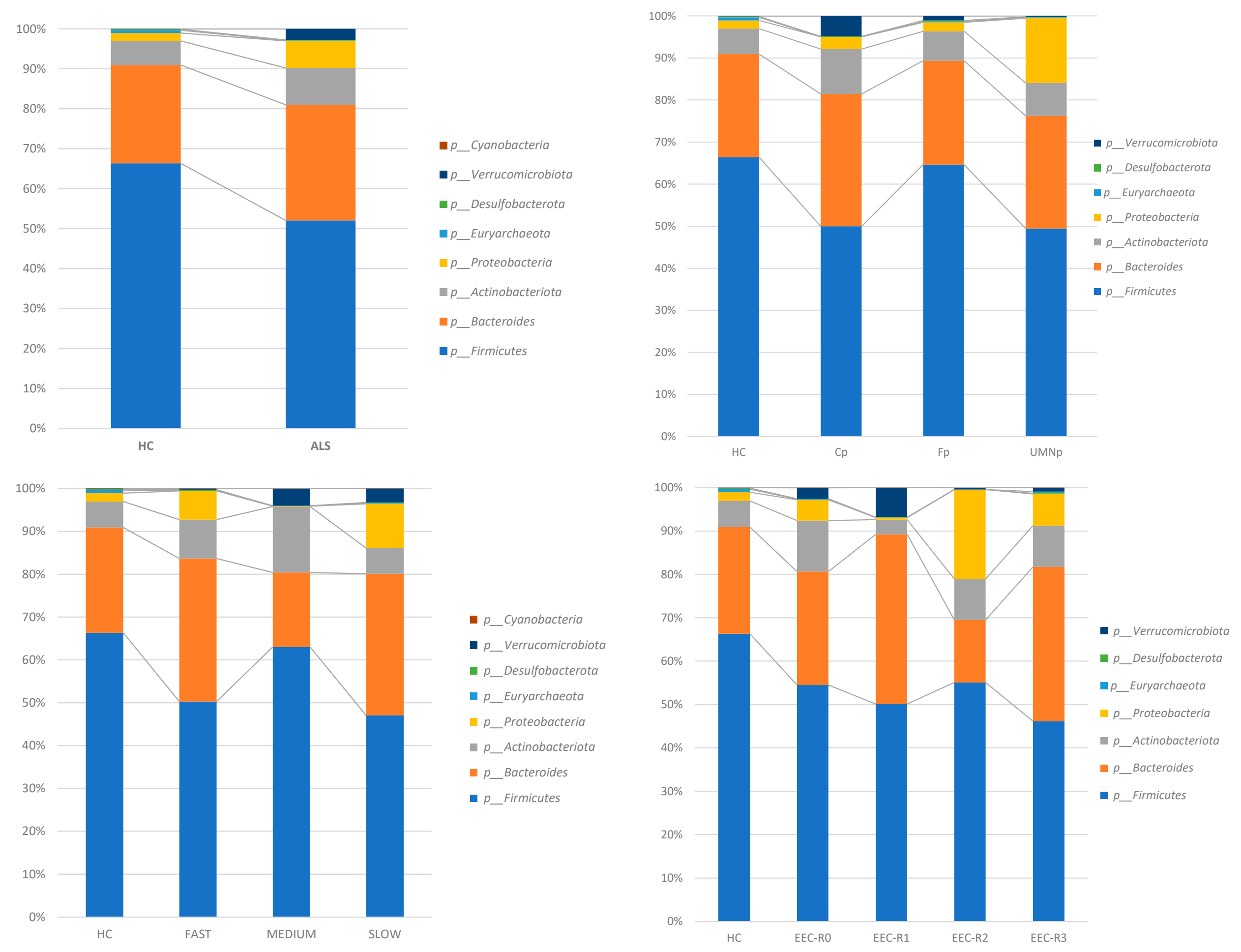

Figure S4. Stacked bar plots displaying the average relative abundance of bacterial amplicon sequence variants (ASVs) identified at the phylum taxonomic level in the gut microbiota of ALS patients' subgroups and healthy controls. Abbreviations are as explained in Figure S1.

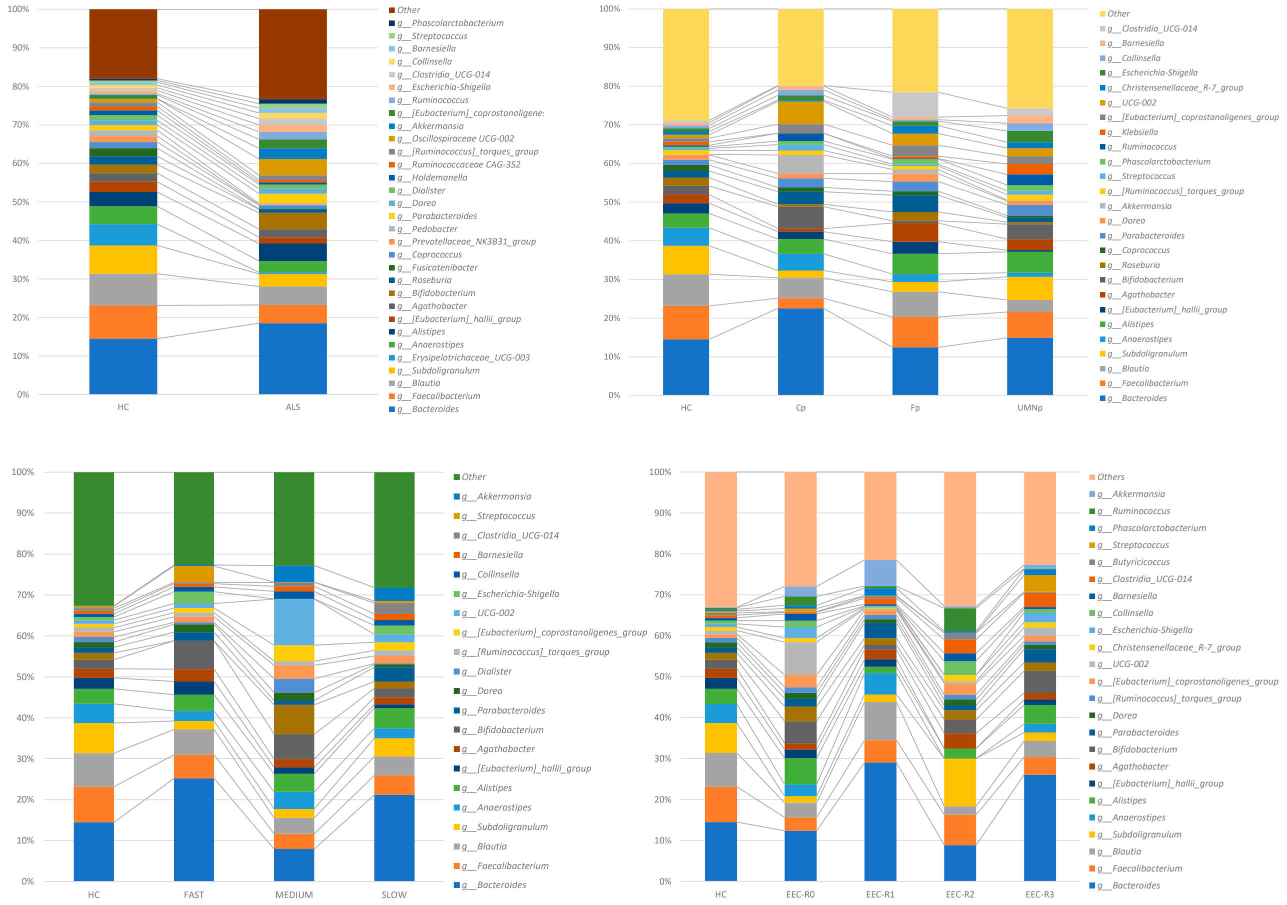

Figure 5S. Stacked bar plots displaying the average relative abundance of bacterial amplicon sequence variants (ASVs) identified at the genus taxonomic level in the gut microbiota of ALS patients' subgroups and healthy controls. Abbreviations are as explained in Figure S1.
